# Perspectives on the HIV continuum of care among adult opioid users in New York City: a qualitative study

**DOI:** 10.1186/s12954-019-0329-z

**Published:** 2019-10-12

**Authors:** Babak Tofighi, Selena S. Sindhu, Chemi Chemi, Crystal Fuller Lewis, Victoria Vaughan Dickson, Joshua D. Lee

**Affiliations:** 1 Department of Population Health, New York University School of Medicine, 180 Madison Avenue, New York, NY 10016 USA; 2 Division of General Internal Medicine and Clinical Innovation, New York University School of Medicine, 180 Madison Avenue, New York, NY 10016 USA; 3 Center for Drug Use and HIV Research (P30DA011041), 180 Madison Avenue, New York, NY 10016 USA; 4 Department of Psychiatry, New York University School of Medicine, New York, USA; 5 The Nathan S. Kline Institute for Psychiatric Research, Orangeburg, USA; 6 NYU Meyers College of Nursing, New York, USA

**Keywords:** HIV, Opioid use disorder, Primary health care, Drug users

## Abstract

**Background:**

Engagement in the HIV care continuum combined with office-based opioid treatment remains a cornerstone in addressing the intertwined epidemics of opioid use disorder (OUD) and HIV/AIDS. Factors influencing patient engagement with OUD and HIV care are complex and require further study.

**Methods:**

In this qualitative study, in-depth interviews were conducted among 23 adult patients who use drugs (PWUD) in an inpatient detoxification program in New York City. The semi-structured interview guide elicited participant experiences with various phases of the HIV care continuum, including factors influencing access to HIV care, interactions with HIV and primary care providers, preferences around integrated care approaches for OUD and HIV, and barriers experienced beyond clinical settings which affected access to HIV care (e.g., insurance issues, transportation, cost, retrieving prescriptions from their pharmacy). Data collection and thematic analysis took place concurrently using an iterative process-based established qualitative research method.

**Results:**

Respondents elicited high acceptability for integrated or co-located care for HIV and OUD in primary care. Factors influencing engagement in HIV care included (1) access to rapid point-of-care HIV testing and counseling services, (2) insurance coverage and costs related to HIV testing and receipt of antiretroviral therapy (ART), (3) primary care providers offering HIV care and buprenorphine, (4) illicit ART sales to pharmacies, (5) disruption in supplies of ART following admissions to inpatient detoxification or residential treatment programs, (6) in-person and telephone contact with peer support networks and clinic staff, (7) stigma, and (8) access to administrative support in primary care to facilitate reengagement with care following relapse, behavioral health services, transportation vouchers, and relocation from subsidized housing exposing patients to actively using peers.

**Conclusion:**

These findings suggest expanding clinical and administrative support in primary care for PWUDs with patient navigators, case managers, mobile health interventions, and peer support networks to promote linkage and retention in care.

## Introduction

The global strategy to eliminate AIDS relies on the 90-90-90 treatment goals ensuring that 90% of all individuals living with HIV know their HIV status, 90% of those are receiving antiretroviral therapy (ART), and 90% of those have attained viral suppression [1]. Progress towards achieving these targets for 2020 relies on the five-stage HIV care continuum, including diagnosis of HIV, initiation on ART and linkage to HIV care, retention in treatment, and optimal viral suppression [1].

The worsening opioid use disorder (OUD) epidemic has ushered in a rise in injection drug use and new HIV and HCV outbreaks [2, 3]. Additionally, numerous studies have described attrition along the HIV care continuum among HIV-infected drug users, including suboptimal HIV testing, delayed entry into HIV care [4, 5], and initiation of ART typically in advanced stages of disease [6]. Barriers in linking to HIV care among people who use drugs (PWUDs) are influenced by numerous patient-, provider-, and systems-level factors, including lack of insurance coverage, access to medications for addiction treatment (MAT), substance use, criminal-justice system involvement, and stigma [7, 8]. Additional patient barriers have included food insecurity, side effects attributed to ARTs, low self-efficacy, limited social support, and lack of rapport and confidence in healthcare providers [9, 10]. Provider-level factors cited by patients in qualitative studies influencing ART adherence included access to clinicians expressing empathy, and access to education and counseling on medication adherence [11]. Systemic influences to reduced access to ART among PWUDs include discrimination of PWUDs by law enforcement, stigma in healthcare systems, and limited access to HIV providers [10, 12].

One strategy to reducing barriers to HIV care for populations with OUD includes the scale-up of integrated or co-located care models that combine medications for addiction treatment (e.g., buprenorphine-naloxone, extended-release naltrexone) with HIV treatment [5, 8, 13]. Primary care approximates a patient-centered care model to effectively reduce the burden of OUD and related comorbidities (e.g., HIV, hepatitis C) by integrating access to addiction pharmacotherapies (e.g., buprenorphine, extended-release naltrexone), ART, patient education, adherence counseling, and access to specialty care [14, 15]. Some prior qualitative studies have described mostly favorable findings among patients enrolled in primary care for OUD and HIV due to convenience, improved patient-provider relationships, treatment adherence, shared power and responsibility in their clinical care, and reinforcement of recovery and relapse prevention due to prioritization of a harm reduction approach among providers [16, 17].

However, further studies are needed to assess the complex interplay of individual experiences within various facets of the HIV care cascade among PWUDs, particularly access to HIV testing, rapid initiation of ART following a positive diagnosis, and reengagement to care in the event of relapse or other unanticipated events [1, 8, 11, 13]. In this qualitative study, we sought to understand experiences among individuals admitted to inpatient detoxification for OUD regarding the HIV care cascade and long-term engagement with integrated primary care treatment of HIV and OUD.

## Methods

### Setting and recruitment

Between January and February 2018, 23 adults 18 years of age and older after their admission in Bellevue Hospital’s inpatient detoxification unit in New York City were recruited by the research team and enrolled in this study [18]. The unit offers detoxification for patients with severe alcohol, opioid, and/or benzodiazepine use disorder and linkage to primary care, specialty addiction treatment, or long-term residential care post-discharge facilitated by unit social workers. Opioid detoxification with methadone or buprenorphine typically lasts between 3 and 5 days. Unit physicians also encourage patients with OUD to consider induction to buprenorphine, extended-release naltrexone, or methadone with referrals to outpatient programs in Bellevue Hospital. A purposive sampling framework was used to select a sample with diverse demographic (e.g., insurance access, race, gender, housing status) and clinical (e.g., HIV and HCV status, poly-substance use) characteristics. Inclusion criteria included (1) diagnosis of OUD and (2) 18 years of age or older. Participants were excluded from the study if they presented with suicidal ideations, cognitive dysfunction, or acute withdrawal symptoms requiring management by unit clinicians. Participants were reimbursed with a transportation voucher at the time of discharge. The study was approved by the New York University School of Medicine Institutional Review Board.

### Data collection and analysis

Participants completed a baseline questionnaire that included demographic characteristics, opioid use history, and healthcare utilization. These data were used to describe the sample. Individual audio-taped interviews were conducted by the principal investigator (BT) and research coordinator (SSS) in a private room located within the inpatient unit. Interviews on average lasted from 1 to 3 h. The semi-structured interview guide was used to elicit participant experiences with various phases of the HIV care continuum, including factors that facilitated access to HIV care, interactions with HIV and primary care providers, preferences around integrated care approaches for OUD and HIV, and barriers experienced beyond clinical settings which affected access to HIV care (e.g., insurance issues, transportation, cost, retrieving prescriptions from their pharmacy). Credibility of participant responses was ensured by asking distinctive questions pertaining to select topics, encouraging participants to share examples to support responses, and use of follow-up questions to address any discrepancies [19].

Audio recordings of interviews were transcribed verbatim, de-identified, verified for accuracy, and analyzed in Dedoose, an online platform for qualitative coding. Study team members (SSS, BT, CC) individually conducted a line-by-line reading of interview transcripts, identified key codes for major topics and ideas, and were then developed into a codebook based on a grounded theory approach. No major inconsistencies emerged in coding. Data collection, analysis, and interpretation took place concurrently through an iterative process-based established qualitative research method [20]. As a result, the codebook was continuously adjusted when new themes were derived from the analysis. Coding schemes were assessed for consistency by the study team using the constant comparison method. Inter-coder reliability was ensured by study team members after transcripts were coded separately and discussed until a consensus was reached on key code findings. Methodological rigor was maintained through the use of memoing, an audit trail of analytic decisions, and peer debriefing [20]. These steps improved the credibility of the findings, allowed the study team to compare coding with an expert in qualitative research (VVD), and incorporated multiple sources for emerging themes.

## Results

The total sample of participants (*n* = 23) were mostly male (78%), the average age was 44 (minimum 21, maximum 62), more than half were African-American (*n* = 8) or Hispanic (*n* = 6), the most elicited past month injection drug use was 78%, and reported severe alcohol was 61% and/or benzodiazepine use disorder was 30% in addition to OUD (100%). Approximately two thirds of participants were HCV-positive (65%, *n* = 13/20) and three participants were never tested or unsure of their HCV status. Eight participants were HIV-positive, most (*n* = 6/8) were adherent to ART and follow-up visits with their HIV provider despite active substance use prior to the interview, and half were also diagnosed with HCV (*n* = 4/8).

Key findings in this qualitative study relating to factors influencing engagement with HIV care included (1) routine access to HIV testing in criminal justice, specialty addiction treatment, and inpatient medical settings; (2) barriers to self-initiated HIV testing due to limited insurance coverage and information regarding voluntary testing sites; (3) frequent in-person and/or telephone support from peers, family, and clinic coordinators were critical in facilitating linkage to HIV and/or buprenorphine providers; (4) incarceration or transferred care between inpatient and residential treatment settings were disruptive to retention in HIV care and adherence; and (5) homelessness, financial insecurity, and exposure to actively using peers exacerbated relapse and/or non-adherence to ART. Stigma related to carrying a diagnosis of HIV and OUD was also expressed by some participants. Lastly, clinicians prescribing methadone or buprenorphine in addition to ART were lauded for reinforcing abstinence and adherence to ART (see Table 1).

**Table 1:** Key themes pertaining to HIV care

Point-of-care HIV testing	“That’s the only time [incarcerated] when I get tested [for HIV]. I do not go to no doctor. Because I be too busy getting high. When I get a place and I’m sober and at a stable mind, maybe I could get tested. When they come around in the jail, and ask me, I am here, why not..”
	“I am not gonna go to no building and get tested because I do not have the time for that. My time would be to get out [active substance use]. If that mobile van is sitting out there, and they telling me that’s what it’s [HIV testing] for, I will go in there. It will ease my consciousness.”
Integrated addiction treatment and HIV care	“I was on suboxone illegally. I was buying it on the streets until I saw my HIV doctor, and I explained to him, my situation that I was buying suboxone in the streets. He told me ‘Why you ain’t tell me? I could prescribe it you.’ So I am like great and I always gave him clean urine. I wasn’t getting high as much.”
	“It was easy because the methadone program I was in, they was helping me with [HIV] treatment as soon as possible. They wanted to get me into it.”
Non-adherence to ART	“It’s like just not a caring attitude, I am here, and my medicine over there. Just like that because I am running. I am in this city, that town, Baltimore, might be in Jersey, Georgia, to New York in like a 8 days span.”
Stigma related to HIV	“… I had a great job. I was making $23 an hour. My supervisor sees me at the locker one day and I was taking my medication. And I guess she thought I was doing drugs so she was like what you doing. I said I am taking my medications, retrovirals. And after that they started treating me different at work. And so I lost my job. They did not say I lost it [employment] because I have HIV, but they found other reasons.”
	“Yes, a lot of people will not admit it [HIV status]. Like my HIV, I have been celibate for 5 years. If I have sex with anyone, I will have to tell that person, listen, this is what I have. But I am not ready to expose myself to anybody. So, I just leave it alone.”
Stigma related to OUD	“With the case manager in the building [HIV/AIDS Service Administration Supportive Housing], she do not really care about nothing, you know. She is just there to maintain the building. Instead of trying to help me with the problem [OUD], she tried to downgrade me, you know. She told me, ‘well I know you guys are getting high here in the building, and you guys got to do something because we are going to start cleaning the house and this and that.’ Instead of saying ‘let me speak to you, you gotta problem, talk to me, you getting high, talk to me, can I help you in anyway?’ She do not do that.”
	“When I came to the emergency room, she [nurse] asked me for my ID. I said I do not have no ID because somebody stole it. She asked me, why I came here? I explained [for detoxification from opioids]. She tried to act like she do not want to believe me. That makes me mad. Trying to make me feel like I’m lying to you. That’s why a lot of people do not go to detox. They die in the street instead of coming to the detox and not be humiliated like that.”
Medicaid restrictions to clinical care	“My Medicaid, this was the big problem. I told them that hospital’s been closed for 2 years. I do not understand why I’ve been restricted to a hospital that’s closed.”
	“My doctor is restricted to [specific Medicaid plan] I’m restricted to one pharmacy on [location], and now that I live in [distant location from pharmacy], it’s a problem.”

### HIV testing experiences

All respondents were amenable to HIV testing even if they were concerned or anxious by a potentially positive test result. Most identified a parent, sibling, or friend who were diagnosed with HIV and actualized the importance of HIV testing and harm reduction practices. One participant expressed high acceptability for HIV testing and avoiding injection drug use due to the death of his HIV-positive brother:

When they came and asked [for HIV testing], there was no hesitation about that. I wanted that done. I had a brother that died with that [HIV infection]. He shared his needles. That's why when I was doing my heroin, I never used needles. That was a big experience.

However, few individuals voluntarily sought HIV testing and emphasized the importance of convenient point-of-care testing (e.g., prisons, emergency rooms, mobile vans), particularly during periods of active drug use (see Table 1). In addition, ongoing engagement with methadone treatment programs (MTP), harm reduction programs, and a community center dedicated to MSM health needs offered routine HIV testing with same-day results. One respondent recalled the benefits of receiving HIV testing and prevention information in a community center dedicated to MSM rather than primary care visits due to the stigma associated with disclosing his sexual orientation to his providers:

… Because of the program [Center], yes [obtaining HIV tests]. Otherwise, I don't think I probably would. I mean I know my status as of three or four months ago. But yeah, it's important to get tested. I also worked at the [Center], which is HIV proactive, so I'm personally more actively educated to do that more than usual.

Unanticipated deactivation of insurance coverage, lack of coverage for HIV testing, limited awareness of HIV/AIDS Service Administration benefits, and restrictions to non-existent or remotely located pharmacies and clinics were major obstacles to HIV testing. Insurance coverage deactivation was attributed to frequent changes in home addresses, lack of notification or correspondences from Medicaid insurance providers, or being unaware of their insurance plan’s expiration date. One participant recalled difficulties in obtaining HIV testing in primary care due to their insurance plan:

Some doctors would say this test will cost money. ‘Your insurance may not cover it. They may cover it, they may not.’ We weren’t sure on that because I have a managed care Medicaid and I chose UnitedHealth thinking it was a good one. It turns out it’s not a great company.

Another participant shared her mother’s experience with limited insurance coverage for her monthly supplies of ART and how:

She’s always complaining because she’s a working person and she’s set a portion of her pay for parts of her medication [ART] cause of her insurance.

Among respondents seeking HIV testing, several reported delays in notification of HIV test results and the shock experienced after notification of a positive result. Suggestions for improving the HIV testing experience included rapid notification of test results and more frequent contact with healthcare staff until they are notified of their test results. One participant emphasized the importance of reducing the anxiety associated with HIV testing with frequent contact with healthcare staff, particularly during periods of active illicit substance use or withdrawal symptoms:

When you wait until they tell you yes or no [HIV results], that's like a hundred years for me. I don't like to feel like that. Apart from that, If I'm using drugs outside, I don't want to be waiting [for HIV test results], especially if I'm sick [withdrawal symptoms]. But I’d be happy speaking with somebody [clinic staff]. I prefer that than being scared waiting.

### Linkage and retention in HIV care

Participants commonly reported delayed access to HIV providers and initiation of ART. Referrals between multiple healthcare providers would take place prior to initiating ART. Among individuals already receiving ART, entry in criminal justice settings, inpatient detoxification, or combination treatment with direct acting antiretrovirals (DAAs) for hepatitis C infection would frequently result in discontinuation or delays in providing ART. One respondent was emphatic about the importance of a single primary care HIV provider that could ensure continuity of care:

Just get me set on a regimen [ART] that I can follow and be consistent and having a doctor that I can talk to and whenever he needs me to come in for a test.

Linkage to residential treatment programs after inpatient detoxification was preferred among some respondents following prolonged periods of illicit substance. Participants were concerned about the impact of reduced cognitive functioning, relapse, and non-adherence to ART. Thus, admission to residential treatment was perceived as an opportunity to improve one’s physical health and cognitive functioning and subsequently “have a clearer head of what I wanna do when I get out of here.” However, participants were frequently concerned about receiving an adequate supply of ART prior to their discharge to aftercare, including residential treatment, due to prior experiences with exhausting supplies of ART and inability to secure refills in various specialty addiction settings.

Additional challenges to ART adherence included illicit purchases by pharmacies of participant’s supplies of ART. Participants expressed motivation to sell their monthly supplies of ART to local pharmacies during periods of active substance use. One participant recalled discretely receiving cash with their monthly refills of ARTs from their pharmacist to incentivize ongoing utilization of their location for subsequent refills:

What they do is when they give you your medication, they’ll put the money [$100] in the bag so nobody could see what’s going on.

All HIV-positive respondents reported receipt of HIV/AIDS Service Administration (HASA) benefits, including subsidized housing and food stamps. However, exposure to actively using peers and drug dealers in HASA subsidized housing exacerbated relapse and nonadherence to ART in some respondents:

Where I live at now [HASA housing], there’s drugs there! It’s right there in the building and people [active users] knock on my door. As soon as I come in the building, I know they’re [active users] gonna keep me company.

### Integrated care for HIV and OUD

Challenges associated with ART adherence following linkage with HIV primary care included limited access to providers and pharmacies after work or during weekend hours, travel, and transportation costs. ART adherence was also complicated during periods of depression and suicidal ideations and lack of access to behavioral health specialists in primary care settings. One participant who had endured several months of worsening depression symptoms recalled:

All they [HIV Provider] do is supply me with my medical treatment for HIV or give me metro cards. They will recommend me to mental health, and do it on the computer, sending out a memo, and tell me they gonna call me [appointment for mental health]. But no one calls me.

Another participant recalled the inability to secure mental health services in his HIV primary care clinic for ongoing depression symptoms which resulted in his relapse to illicit opioids and selling supplies of ART to sustain ongoing substance use:

In the beginning, I was really motivated. After a while, the depression sank in. I started using more drugs. You know, I went to my doctor [HIV provider] when I had to. Sometimes I missed it [follow-up visit]. I sold my medications sometimes, my HIV medications.

Frequent communication with HIV clinic staff was noted for resolving a range of clinical and administrative needs that may have jeopardized adherence to ART and MAT. One participant recalled how frequent contact by his HIV clinic was perceived as highly useful in securing linkage to addiction treatment or transportation. Importantly, calls by clinic staff, even during periods of active substance use, were helpful when the patient was contemplating reinitiating clinic visits and ART following an extended period of illicit substance use:

I have a lady there [HIV primary care program]. She has been trying to reach me for a while... She helps me with whatever issue I have, if I need to get into a drug program, help with taking me somewhere, you know, stuff like that.

Adherence strategies for ARTs during periods of extended substance use included taking medications during the evening after episodes of substance use when patients were less likely to be soliciting or using heroin. Suggestions for improved adherence to ART and primary care visits included telephone call or text message reminders, having access to lab results (e.g., viral loads, CD4 count), utilizing pharmacies that were open during evening and weekend hours, obtaining refills from pharmacies located in proximity to HIV primary care clinics, and encouraging patients to link friends and family members requiring HIV care and MAT to their clinic. The need for peer support during initial engagement with an HIV primary care clinic was also described by one participant:

I came in there [HIV primary care] blind but I felt very comfortable going there with a friend of mine who is HIV positive and was also a patient there.

Lastly, some respondents requested further education regarding potential interactions between ARTs and medications for addiction treatment (e.g., methadone, buprenorphine, extended-release naltrexone). One participant attributed worsening withdrawal symptoms following initiation of ART due to:

The HIV medicine ate up the methadone. I would have to keep upping my dose [methadone] but no matter how much I would up my dose, I had to get off that and get on suboxone.

## Discussion

Findings emerging from this qualitative study suggest mostly positive experiences with routine HIV testing in criminal justice and healthcare settings. However, barriers to self-initiated HIV testing persist (e.g., lack of insurance coverage or information regarding voluntary testing sites). Most participants were favorable to integrated treatment for HIV and OUD in primary care. Key insights shared by participants emphasized enhancing patient-centered approaches (e.g., shared decision making, emotional support, coordination and integration of care, information to facilitate health promotion) that reinforced key elements of the HIV care cascade (i.e., testing, diagnosis, receipt and retention on ART and medical care, viral suppression) [17, 21, 22]. Such approaches were critical in reengaging individuals with HIV care across different clinical settings (e.g., emergency rooms, inpatient detoxification, residential treatment).

Suggestions for ensuring retention in HIV care during periods of active substance use included a harm reduction approach combined with frequent patient-physician communication, peer and family involvement in care, and timely access to behavioral health services. Lastly, participants stressed the challenges experienced with reengaging or adhering to HIV and OUD treatment care plans in an increasingly complex and fragmented healthcare delivery system, particularly with unanticipated insurance and HASA-related issues (e.g., restrictions on subsidized housing, clinics, and/or pharmacies) [5, 13, 16, 17, 21].

### HIV testing and initiation of ART

Routine HIV testing was readily available in criminal justice, specialty addiction, and tertiary healthcare settings (e.g., emergency rooms, inpatient detoxification). However, challenges persisted with self-initiated HIV testing reflecting similar findings among PWUDs nearly two decades ago, including inadequate insurance coverage, limited information regarding voluntary testing sites, and anxiety associated with delayed notification of test results [23, 24]. Peer support networks, harm reduction programs, mobile vans, and rapid HIV testing were distinguished for reducing the anxiety and barriers associated with HIV testing. These findings are also complemented by recent studies demonstrating improved rates of HIV testing among high-risk populations using home-based HIV testing kits [25], vending machines dispensing HIV self-test kits [26], text message reminders, sexual networking apps and online forums, and social media (e.g., Facebook) [27, 28].

Although barriers persist to ART coverage [7], interviews yielded mostly positive experiences with initiation and adherence to ART. The relative ease of access to ART was attributed to factors reinforced by evidence-based practice, including the simplicity and effectiveness of combining ART with effective pharmacotherapies for OUD in primary care and specialty addiction treatment settings, receipt of ART was not contingent upon demonstrating abstinence from opioids, and frequent contact with clinic staff to resume treatment in the event of a missed visit or relapse [13, 29]. Lastly, participant experiences in integrated care (i.e., receipt of buprenorphine and ART) reinforce prior study findings that relapse to illicit substances did not appear to contribute to loss to follow-up with both HIV and buprenorphine [16, 17].

More nuanced elements of patient-centered care were emphasized by participants to undergird optimal ART initiation and adherence, including frequent in-person and/or telephone contact with clinic staff, timely linkage with specialty care, accompanying actively using peers to their initial visit, and empathetic encounters with clinic staff knowledgeable in treating OUD [30–32]. Such attributes of primary care staff were perceived as essential in addressing unanticipated clinical issues (e.g., withdrawal symptoms, interactions between ART and opioid agonist therapy, relapse and reinitiation of ART, depression symptoms, suicidal ideations, transportation to clinics) that echo prior qualitative studies among individuals with OUD and HIV [5–7, 13, 32, 33]. Low-cost strategies, such as HIV provider training in patient-centered communication, buprenorphine treatment, telephone access to interdisciplinary addiction specialists, and improved reimbursement for primary care providers treating HIV and OUD offer novel approaches to improving retention in care and clinical outcomes [13, 34–36].

Although participants did not directly express interest in deferring non-clinical issues to their primary care team (e.g., relocation from HASA subsidized housing units with active drug use, placement in sober housing, insurance restrictions), expanding HIV administrative support (e.g., case management, social work) in primary care may offset much of the fragmented healthcare delivery experience of HIV-positive populations with OUD and potentially improve retention. The integration of administrative support within primary care for populations with HIV has been reinforced by over two decades of funding initiatives (e.g., Federal Substance Abuse Block Grant, Ryan White CARE Act) and research in improving engagement with HIV care, particularly among uninsured, women, PWUD, and younger adults [37]. However, larger-scale adoption of administrative support for individuals with HIV has been inconsistent [38, 39].

Among the spectrum of clinical needs required by participants, access to behavioral health services appeared to be the most problematic. These findings echo prior studies describing high rates of untreated psychiatric symptoms among HIV-positive PWUDs, relapse and nonadherence to ART, and discordant mental health services in HIV primary care and among individuals with OUD [40, 41]. The provision of behavioral health resources in co-located or fully integrated models of primary care for PWUD have been described previously to be associated with increased survival [8] and reinforces the supportive role of office-based settings in prioritizing the patient-provider therapeutic alliance, communication, and timely coordination to mental health services [17, 42].

Lastly, epidemiological modeling studies evaluating incomplete engagement in HIV care have identified interventions enhancing retention and reengagement as the most cost-effective, yet understudied approach to improve the HIV care cascade [43]. In addition to frequent telephone contact with clinic staff, prior findings by this study team among inpatient detoxification patients have described high rates of acceptability of adopting technology-based interventions (e.g., text messaging, smartphone apps, social media) to enhance engagement with effective pharmacotherapies for substance use disorders, HIV, and related co-morbidities in primary care (e.g., HCV, depression, smoking cessation) [28]. Thus, technology and peer-driven interventions offer low-cost approaches to offset gaps in administrative and clinical support for vulnerable patient populations with HIV and substance use disorders in primary care settings [44].

### Limitations

Limitations to this study’s findings include (1) the small sample size recruited from an urban, publicly funded, inpatient detoxification program; (2) all participants had relapsed to opioid use following periods of abstinence or engagement with methadone or buprenorphine maintenance treatment; (3) criticisms of HASA and ER staff should be interpreted with caution given the very limited sample of participants who expressed these experiences; and (4) participants were not evaluated based on pertinent demographic characteristics (i.e., age, education level, employment status) and clinical characteristics (i.e., substance use patterns, psychiatric history).

## Conclusions

Despite improved access to rapid point-of-care HIV testing, ART, and favorable experiences with primary care providers prescribing ART and buprenorphine among individuals with OUD, additional resources are needed to address barriers to optimal linkage and retention in primary care for PWUD with HIV. Future strategies should consider expanding social service, patient navigators, and mental health professionals in primary care to ensure optimal engagement with HIV care. Illicit practices by retail pharmacies to solicit HIV-positive patients to sell monthly supplies of ART or receive cash with monthly prescriptions highlight manipulative practices undermining fundamental ethical principles and exacerbating health disparities among marginalized populations with HIV. In addition, participants that did not have access to HIV providers prescribing buprenorphine experienced delays in receiving treatment for OUD resulting in extended periods of illicit substance use and nonadherence to ART.

## Data Availability

Participants completed a baseline questionnaire that included demographic characteristics, opioid use history, and healthcare utilization. These data were used to describe the sample. Individual audio-taped interviews were conducted by the principal investigator (BT) and research coordinator (SSS) in a private room located within the inpatient unit. Interviews on average lasted from 1 to 3 h. Audio recordings of interviews were transcribed verbatim, de-identified, verified for accuracy, and analyzed in Dedoose, an online platform for qualitative coding.
